# Fast Compression of MCMC Output

**DOI:** 10.3390/e23081017

**Published:** 2021-08-06

**Authors:** Nicolas Chopin, Gabriel Ducrocq

**Affiliations:** Institut Polytechnique de Paris, ENSAE Paris, CEDEX, 92247 Malakoff, France; gabriel.ducrocq@ensae.fr

**Keywords:** control variates, Markov chain Monte Carlo, thinning

## Abstract

We propose cube thinning, a novel method for compressing the output of an MCMC (Markov chain Monte Carlo) algorithm when control variates are available. It allows resampling of the initial MCMC sample (according to weights derived from control variates), while imposing equality constraints on the averages of these control variates, using the cube method (an approach that originates from survey sampling). The main advantage of cube thinning is that its complexity does not depend on the size of the compressed sample. This compares favourably to previous methods, such as Stein thinning, the complexity of which is quadratic in that quantity.

## 1. Introduction

MCMC (Markov chain Monte Carlo) remains, to this day, the most popular approach to sampling from a target distribution *p*, in particular in Bayesian computations [1].

Standard practice is to run a single chain, $X_{1},\ldots ,X_{N}$ according to a Markov kernel that leaves invariant *p*. It is also common to discard part of the simulated chain, either to reduce its memory footprint, or to reduce the CPU cost of later post-processing operations, or more generally for the user’s convenience. Historically, the two common recipes for compressing an MCMC output are:

- burn-in, which allows discarding the *b* first states;
- thinning, which allows retaining only one out of *t* (post burn-in) states.

The impact of either recipes on the statistical properties of the subsampled estimates are markedly different. Burn-in reduces the bias introduced by the discrepancy between *p* and the distribution of the initial state $X_{1}$ (since $X_{b}\approx p$ for *b* large enough). On the other hand, thinning always increases the (asymptotic) variance of MCMC estimators [2].

Practitioners often choose *b* (the burn-in period) and *t* (the thinning frequency) separately, in a somewhat ad hoc fashion (i.e., through visual inspection of the initial chain), or using convergence diagnosis such as, e.g., those reviewed in [3].

Two recent papers [4,5] cast a new light on the problem of compressing an MCMC chain by considering, more generally, the problem, for a given *M*, of selecting the subsample of size *M* that best represents (according to a certain criterion) the target distribution *p*. We focus for now on [5], for reasons we explain below.

Stein thinning, the method developed in [5], chooses the subsample S of size *M* which minimises the following criterion:

(1) $$D\left(S\right):=\frac{1}{M^{2}}\sum_{m,n\in S}k_{p}\left(X_{m},X_{n}\right),\;S\subset \left\{1,\ldots ,N\right\},\;\left|S\right|=M$$

where $k_{p}$ is a *p*-dependent kernel function derived from another kernel function *k*: X×X→R, as follows:

$$\begin{array}{c} k_{p}\left(x,y\right)=\nabla_{x}\cdot \nabla_{y}k\left(x,y\right)+\langle \nabla_{x}k\left(x,y\right),s_{p}\left(y\right)\rangle +\langle \nabla_{y}k\left(x,y\right),s_{p}\left(x\right)\rangle +k\left(x,y\right)\langle s_{p}\left(x\right),s_{p}\left(y\right)\rangle \end{array}$$

with 〈·,·〉 being the Euclidean inner product, $s_{p}\left(x\right):=\nabla \log p\left(x\right)$ is the so-called score function (gradient of the log target density), and ∇ is the gradient operator.

The rationale behind criterion (1) is that it may be interpreted as the KSD (kernel Stein discrepancy) between the true distribution *p* and the empirical distribution of subsample *S*. We refer to [5] for more details on the theoretical background of the KSD, and its connection to Stein’s method.

Stein thinning is appealing, as it seems to offer a principled, quasi-automatic way to compress an MCMC output. However, closer inspection reveals the following three limitations.

First, it requires computing the gradient of the log-target density, $s_{p}\left(x\right)=\nabla \log p\left(x\right)$. This restricts the method to problems where this gradient exists and is tractable (and, in particular, to $X=R^{d}$).

Second, its CPU cost is $O(NM^{2})$. This makes it nearly impossible to use Stein thinning for M≫100. This cost stems from the greedy algorithm proposed in [5], see their Algorithm 1, which adds at iteration *t* the state $X_{i}$ which minimises $k_{p}\left(X_{i},X_{i}\right)+\sum_{j\in S_{t-1}}k_{p}\left(X_{i},X_{j}\right)$, where $S_{t-1}$ is the sample obtained from the t−1 previous iterations.

Third, its performance seems to depend in a non-trivial way on the original kernel function *k*; the authors of [5] propose several strategies for choosing and scaling *k*, but none of them seem to perform uniformly well in their numerical experiments.

We propose a different approach in this paper, which we call cube thinning, and which addresses these shortcomings to some extent. Assuming the availability of *J* control variates (that is, of functions $h_{j}$ with known expectation under *p*), we cast the problem of MCMC compression as that of resampling the initial chain under constraints based on these control variates. The main advantage of cube thinning is that its complexity is $O(NJ^{3})$; in particular, it does not depend on *M*. That makes it possible to use it for much larger values of *M*. We shall discuss the choice of *J*, but, by and large, *J* should be of the same order as *d*, the dimension of the sampling space. The name stems from the cube method of [6], which plays a central part in our approach, as we explain in the body of the paper.

The availability of control variates may seem like a strong requirement. However, if we assume we are able to compute $s_{p}\left(x\right)=\nabla \log p\left(x\right)$, then (for a large class of functions $\phi :R^{d}\to R^{d}$, which we define later)

$$E_{p}\left[\phi \left(x\right)s_{p}\left(x\right)+\nabla_{x}\cdot \phi \left(x\right)\right]=0$$

where $\nabla_{x}\cdot \phi$ denotes the divergence of ϕ. In other words, the availability of the score function implies, automatically, the availability of control variates. The converse is not true: there exists control variates, e.g., [7], that are not gradient-based. One of the examples we consider in our numerical examples feature such non gradient-based control variates; as a result, we are able to apply cube thinning, although Stein thinning is not applicable.

The supporting methods of [4] do not require control variates. It is thus more generally applicable than either cube thinning or Stein thinning. On the other hand, when gradients (and thus control variates) are available, the numerical experiments of [5] suggest that Stein thinning outperforms support points. From now on, we focus on situations where control variates are available.

This paper is organised as follows. Section 2 recalls the concept of control variates, and explains how control variates may be used to reweight an MCMC sample. Section 3 describes the cube method of [6]. Section 4 explains how to combine control variates and the cube method to perform cube thinning. Section 5 assesses the statistical performance of cube thinning through two numerical experiments.

We use the following notations throughout: *p* denotes both the target distribution and its probability density; p(f) is a short-hand for the expectation of f(X) under *p*. The gradient of a function *f* is denoted by $\nabla_{x}f\left(x\right)$, or simply ∇f(x) when there is no ambiguity. The i-th component of a vector $v\in R^{d}$ is denoted by v[i], and it is transposed by $v^{t}$. The vectors of the canonical basis of $R^{d}$ are denoted by $e_{i}$, i.e., $e_{i}\left[j\right]=1$ if j=i, 0 otherwise. Matrices are written in upper-case; the kernel (null space) of matrix *A* is denoted by kerA. The set of functions $f:\Omega \to R^{d}$ that are continuously differentiable is denoted by $C^{1}\left(\Omega ,R^{d}\right)$.

## 2. Control Variates

### 2.1. Definition

Control variates are a very well known way to reduce the variance of Monte Carlo estimates—see, e.g., the books of [1,8,9].

Suppose we want to estimate the quantity $p\left(f\right)=E_{p}\left[f\left(X\right)\right]$ for a suitable $f:R^{d}\to R$, based on an IID (independent and identically distributed) sample $\left\{X_{1},\cdots ,X_{N}\right\}$ from distribution *p*. The generalisation of control variates to MCMC will be discussed in Section 4.

The usual Monte Carlo estimator of p(f) is

(2) $$\hat{p}\left(f\right)=\frac{1}{N}\sum_{n=1}^{N}f\left(X_{n}\right).$$

Assume we know $J\in N^{⋆}$ functions $h_{j}:R^{d}\to R$ for j∈{1,⋯,J} such that $p(h_{j})=0$. Functions with this property are called control variates. We can use this property to build an estimate with a lower variance: let us denote $h\left(X\right)={\left(h_{1}\left(X\right),\cdots ,h_{J}\left(X\right)\right)}^{t}$ and write our new estimate:

(3) $${\hat{p}}_{\beta }\left(f\right)=\frac{1}{N}\sum_{n=1}^{N}f\left(X_{n}\right)+\beta^{t}h\left(X_{n}\right)$$

with $\beta \in R^{J}$. Then it is straightforward to show that $E\left[{\hat{p}}_{\beta }\left(f\right)\right]=E\left[\hat{p}\left(f\right)\right]=p\left(f\right)$. Depending on the choice of β, we may have $\mathrm{Var}\left[{\hat{p}}_{\beta }\left(f\right)\right]\leq \mathrm{Var}\left[\hat{p}\left(f\right)\right]$. The next section discusses how to choose such a β.

### 2.2. Control Variates as a Weighting Scheme

The standard approach to choose β consists of two steps. First, one shows easily that the value the minimises the variance of estimator (3) is:

(4) $$\beta^{⋆}\left(f\right)=\mathrm{Var}{\left(h\left(X\right)\right)}^{-1}\mathrm{Cov}\left(h\left(X\right),f\left(X\right)\right)$$

where Var(h(X)) is the J×J variance matrix of the vector h(X) and Cov(h(X),f(X)) is the J×1 vector such that $\mathrm{Cov}{\left(h\left(X\right),f\left(X\right)\right)}_{i,1}=\mathrm{Cov}\left(f\left(X\right),h_{i}\left(X\right)\right)$.

Second, one realises that this quantity may be estimated from the sample $X_{1},\ldots ,X_{N}$ through a simple linear regression model, where the $f(X_{n})$s are the outcome, and the $h_{j}\left(X_{n}\right)$s are the predictors:

(5) $$f\left(X_{n}\right)\approx \mu +\beta^{t}h\left(X_{n}\right)+\epsilon_{n},\;E\left[\epsilon_{n}\right]=0.$$

More precisely, let $\gamma \in R^{J+1}$ be the vector such that $\gamma^{t}=\left(\mu ,\beta^{t}\right)$, $H=(H_{ij})$ the design matrix such that $H_{i1}=1$, $H_{i(j+1)}=h_{j}\left(X_{i}\right)$, and $F=(f\left(X_{1}\right),\ldots ,f\left(X_{N}\right))$. Then, the OLS (ordinary least squares) estimate of γ is

(6) $${\hat{\gamma }}_{\mathrm{OLS}}={\left(H^{t}H\right)}^{-1}H^{t}F.$$

Since $E[f\left(X_{n}\right)]=\mu$ in this artificial regression model, the first component of ${\hat{\gamma }}_{\mathrm{OLS}}$:

(7) $${\hat{p}}_{⋆}\left(f\right):={\hat{\gamma }}_{\mathrm{OLS}}\times e_{1},$$

actually corresponds to estimate (3) when $\beta ={\hat{\beta }}_{\mathrm{OLS}}$.

At first glance, the approach described above seems to require implementing a different linear regression for each function *f* of interest. Ref. [9] noted, however, that one may re-express (7) as a weighted average:

(8) $${\hat{p}}_{⋆}\left(f\right)=\sum_{n=1}^{N}w_{n}f\left(X_{n}\right)$$

where the weights $w_{n}$ sum to one, and do not depend on *f*. It is thus possible to compute these weights once from a given sample (given a certain choice of control variates), and then quickly compute ${\hat{p}}_{⋆}\left(f\right)$ for any function *f* of interest.

The exact expression of the weights are easily deduced from (7) and (6): $w=(w_{n})$ with

$$w=H{\left(H^{t}H\right)}^{-1}e_{1}.$$

### 2.3. Gradient-Based Control Variates

In this section and the next, we recall generic methods to construct control variates. This section specifically considers control variates that are derived from the score function, $s_{p}\left(x\right)=\nabla \log p\left(x\right)$. (We therefore assume that this quantity is tractable.)

Under the following two conditions:

1. The probability density $p\in C^{1}\left(\Omega ,R\right)$ where $\Omega \subseteq R^{d}$ is an open set;
2. Function $\phi \in C^{1}\left(\Omega ,R^{d}\right)$ is such that $\oint_{\partial \Omega }p\left(x\right)\phi \left(x\right)\cdot n\left(x\right)S\left(dx\right)=0$ where $\oint_{\partial \Omega }$ denotes the integral over the boundary of Ω, and S(dx) is the surface element at x∈∂Ω.

The following function:

(9) $$h\left(x\right)=\nabla_{x}\cdot \phi \left(x\right)+\phi \left(x\right)\cdot s_{p}\left(x\right)$$

is a control variate: p(h)=0, see, e.g., [10] or [11] for further details. To gain some insight, note that in dimension 1 and assuming the domain of integration is an interval ]a,b[⊂R, this amounts to an integration by parts with the condition that h(b)p(b)−h(a)p(a)=0.

Thus, whenever the score function is available (and the conditions above hold), we are able to construct an infinite number of control variates (one for each function ϕ). For simplicity, we shall focus on the following standard classes of such functions. First, for i=1,…,d,

$$\begin{array}{cc} \phi_{i}:R^{d} & \to R^{d}\\ x & \mapsto e_{i} \end{array}$$

which leads to the following *d* control variates:

(10) $$h_{i}\left(x\right)=s_{p}\left(x\right)\left[i\right].$$

For a Gaussian target, N(μ,Σ), the score is $s_{p}\left(x\right)=-\Sigma^{-1}\left(x-\mu \right)$, and the control variates above make it possible to reweight the Monte Carlo sample to make it have the same expectation as the target distribution.

Second, we consider, for i,j=1,…,d:

$$\begin{array}{cc} \phi_{ij}:R^{d} & \to R^{d}\\ x & \mapsto x\left[i\right]e_{j} \end{array}$$

which leads to the following $d^{2}$ control variates:

(11) $$h_{ij}\left(x\right)=1\left\{i=j\right\}+x\left[i\right]s_{p}\left(x\right)\left[j\right].$$

Again, for a Gaussian target N(μ,Σ), this makes it possible to fix the empirical covariance matrix to true covariance Σ.

In our simulations, we consider two sets of control variates: the ‘full’ set, consisting of the *d* control variates defined by (10), and the $d^{2}$ control variates defined by (11), and a ‘diagonal’ set of 2d control variates, where for (11), we only consider the cases where i=j. Of course, the former set should lead to a better performance (lower variance), but since the complexity of our approach will be $O(J^{3})$, where *J* is the number of control variates, taking $J=O(d^{2})$ may be too expensive whenever the dimension *d* is large. In fact, when *d* is very large, one might even consider considering only control variates that depend on a few components of *x* of interest.

### 2.4. MCMC-Based Control Variates

We mention in passing other ways to construct control variates, in particular in the context of MCMC.

For instance, [7] noted that, for a Markov chain $\left\{X_{n}\right\}$, the quantity

$$\phi \left(X_{n}\right)-E\left[\phi \left(X_{n}\right)|X_{n=1}\right]$$

has zero expectations. In particular, if the MCMC kernel is a Gibbs sampler, it is likely that one is able to compute the conditional expectation of each component, i.e., ϕ(x)=x[i] for i=1,…,d.

See also [12,13] for other ways to construct control variates when the $X_{n}$s are simulated from a Metropolis kernel.

## 3. The Cube Method

We review in this section the cube method of [6]. This method originated from survey sampling and is a way to sample from a finite population under constraints. The first subsection gives some definitions, the second one explains the flight phase of the cube method and the third subsection discusses the landing phase of the method.

### 3.1. Definitions

Suppose we have a finite population {1,⋯,N} of *N* individuals and that to each individual n=1,…,N is associated a variable of interest $y_{n}$ and *J* auxiliary variables, $v_{n}=\left(v_{n1},\cdots ,v_{nJ}\right)$. Without loss of generality, suppose also that the *J* vectors $\left(v_{1j},\cdots ,v_{Nj}\right)$ are linearly independent. We are interested in estimating the quantity $Y=\sum_{n=1}^{N}y_{n}$ using a subsample of {1,…,N}. Furthermore, we know the exact value of each sum $V_{j}=\sum_{n=1}^{N}v_{nj}$, and we wish to use this auxiliary information to better estimate *Y*.

We assign, to each individual *n*, a sampling probability $\pi_{n}\in \left[0,1\right]$. We consider random variables $S_{n}$ such that, marginally, $P\left(S_{n}=1\right)=\pi_{n}$. We may then define the Horvitz–Thompson estimator of *Y* as

(12) $$\hat{Y}=\sum_{n=1}^{N}\frac{S_{n}y_{n}}{\pi_{n}}$$

which is unbiased, and which depends only on selected individuals (i.e., $S_{n}=1$).

We define similarly the Horvitz–Thompson estimator of $V_{j}$ as

(13) $${\hat{V}}_{j}=\sum_{n=1}^{N}\frac{S_{n}v_{nj}}{\pi_{n}}.$$

Our objective is to construct a joint distribution ξ for the inclusion variables $S_{n}$ such that $P_{\xi }\left(S_{n}=1\right)=\pi_{n}$ for all n=1,…,N, and

(14) $$\hat{V}=V\;\xi -\mathrm{almost}\;\mathrm{surely}.$$

where $V=(V_{1},\ldots ,V_{J})$, $\hat{V}=\left({\hat{V}}_{1},\ldots ,{\hat{V}}_{J}\right)$. Such a probability distribution is called a balanced sampling design.

### 3.2. Subsamples as Vertices

We can view all the possible samples from {1,…,N} as the vertices of the hypercube $C={\left[0,1\right]}^{N}$ in $R^{N}$. A sampling design with inclusion probabilities $\pi_{n}=P_{\xi }\left(S_{n}=1\right)$ is then a distribution over the set of these vertices such that E[S]=π, where $S={\left(S_{1},\ldots ,S_{N}\right)}^{t}$, and $\pi ={\left(\pi_{1},\ldots ,\pi_{N}\right)}^{t}$ is the vector of inclusion probabilities. Hence, π is expressed as a convex combination of the vertices of the hypercube.

We can think of a sampling algorithm as finding a way to reach any vertex of the cube, starting at π, while satisfying the balancing Equation (14). However, before we describe such a sampling algorithm, we may wonder if it is possible to find a vertex such that (14) is satisfied.

### 3.3. Existence of a Solution

The balancing equation, Equation (14), defines a linear system. Indeed, we can re-express (14) as *S*, as a solution to As=V, where $A=(A_{jn})$ is of dimension J×N, $A_{jn}=v_{kn}/\pi_{n}$. This system defines a hyperplane *Q* of dimension N−J in $R^{N}$.

What we want is to find vertices of the hypercube C that also belong to the hyperplane *Q*. Unfortunately, it is not necessarily possible, as it depends on how the hyperplane *Q* intersects cube C. In addition, there is no way to know beforehand if such a vertex exists. Since π∈Q, we know that K:=C∩Q≠∅ and is of dimension N−J. The only thing we can say is stated Proposition 1 in [6]: if *r* is a vertex of K, then in general q=card({n:0<r[n]<1})≤J.

The next section describes the flight phase of the cube algorithm, which generates a vertex in K when such vertices exist, or which, alternatively, returns a point in K with most (but not all) components set to zero or one. In the latter case, one needs to implement a landing phase, which is discussed in Section 3.5.

### 3.4. Flight Phase

The flight phases simulates a process π(t) which takes values in K=C∩Q, and starts at π(0)=π. At every time *t*, one selects a unit vector u(t), then one chooses randomly between one of the two points that are in the intersection of the hypercube C and the line parallel to u(t) that passes through π(t−1). The probability of selecting these two points are set to ensure that π(t) is a martingale; in that way, we have $E[\pi_{t}]=\pi$ at every time step. The random direction u(t) must be generated to fulfil the following two requirements: (a) that the two points are in *Q*, i.e., u(t)∈kerA, and (b) whenever π(t) reaches one of the faces of the hypercube, it must stay within that face; thus, u(t)[k]=0 if π(t−1)[k]=0 or 1.

Algorithm 1 describes one step of the flight phase.
**Algorithm 1:** Flight phase iteration **Input**: π(t−1) **Output**: π(t) ^1^ Sample u(t) in kerA with $u_{k}\left(t\right)=0$ if the *k*-th component of π(t−1) is an integer. ^2^ Compute $\lambda_{1}^{⋆}$ and $\lambda_{2}^{⋆}$, the largest values of $\lambda_{1}>0$ and $\lambda_{2}>0$ such that: $0\leq \pi \left(t-1\right)+\lambda_{1}u\left(t\right)\leq 1$ and $0\leq \pi \left(t-1\right)-\lambda_{2}u\left(t\right)\leq 1$. ^3^ With probability $\lambda_{2}^{⋆}/\left(\lambda_{1}^{⋆}+\lambda_{2}^{⋆}\right)$, set $\pi \left(t\right)\leftarrow \pi \left(t-1\right)+\lambda_{1}u\left(t\right)$; otherwise, set $\pi \left(t\right)\leftarrow \pi \left(t-1\right)-\lambda_{2}u\left(t\right)$.

The flight phase stops when Step 1 of Algorithm 1 cannot be performed (i.e., no vector u(t) fulfils these conditions). Until this happens, each iteration increases by at least one the number of components in π(t) that are either zero or one. Thus, the flight phases completes at most in *N* steps.

In practice, to generate u(t), one may proceed as follows: first generate a random vector $v\left(t\right)\in R^{N}$, then project it in the constraint hyperplane: $u\left(t\right)=I\left(t\right)v\left(t\right)-I\left(t\right)A^{t}{\left(AI\left(t\right)A^{t}\right)}^{-}AI\left(t\right)v\left(t\right)$, where I(t) is a diagonal matrix such that $I_{kk}\left(t\right)$ is 0 if $\pi_{k}\left(t\right)$ is an integer and 1 otherwise, and $M^{-}$ denotes the pseudo-inverse of the matrix *M*.

The authors of [14] propose a particular method to generate vector v(t), which ensures that the complexity of a single iteration of the flight phase is $O(J^{3})$. This leads to an overall complexity of $O(NJ^{3})$ for the flight phase, since it terminates in at most *N* iterations.

### 3.5. Landing Phase

Denote by $\pi^{⋆}$ the value of process π(t) when the flight phase terminates. If $\pi^{⋆}$ is a vertex of C (i.e., all its components are either zero or one), one may stop and return $\pi^{⋆}$ as the output of the cube algorithm. If $\pi^{⋆}$ is not a vertex, this informs us that no vertex belongs to K. One may implement a landing phase, which aims at choosing randomly a vertex which is close to $\pi^{⋆}$, and such that the variance of the components of $\hat{V}$ is small.

Appendix A gives more details on the landing phase. Note that its worst-case complexity is $O(2^{J})$. However, in practice, it is typically either much faster, or not required (i.e., $\pi^{⋆}$ is already a vertex) as soon as J≪N.

## 4. Cube Thinning

We now explain how the previous ingredients (control variates, and the cube method) may be combined in order to thin a Markov chain, $X_{1},\ldots ,X_{N}$, into a subsample of size *M*. As before, the invariant distribution of the chain is denoted by *p*, and we assume we know of *J* control variates $h_{j}$, i.e., $p(h_{j})=0$ for j=1,…,J.

### 4.1. First Step: Computing the Weights

The first step of our method is to use the *J* control variates to compute the *N* weights $w_{n}$, as defined at the end of Section 2.2. Recall that these weights sum to one, and that they automatically fulfil the constraints:

(15) $$\sum_{n=1}^{N}w_{n}h_{j}\left(X_{n}\right)=0$$

for j=1,…,J, and that we use them to compute

(16) $${\hat{p}}_{⋆}\left(f\right)=\sum_{n=1}^{N}w_{n}f\left(X_{n}\right)$$

as a low-variance estimate for p(f) for any *f*.

Recall that the control variates procedure we described in Section 2 assume that the input variables, $X_{1},\ldots ,X_{N}$, are IID. This is obviously not the case in an MCMC context; however, we follow the common practice [10,11] of applying the procedure to MCMC points as if they were IID points. This implies that the weighted estimate above corresponds to a value of β in (3) that does not minimise the (asymptotic) variance of estimator (3). It is actually possible to estimate the value of β that minimises the asymptotic variance of an MCMC estimate [7,15]. However, this type of approach is specific to certain MCMC samplers, and, critically for us, it cannot be cast as a weighting scheme. Thus, we stick to this standard approach.

**Figure 1:**
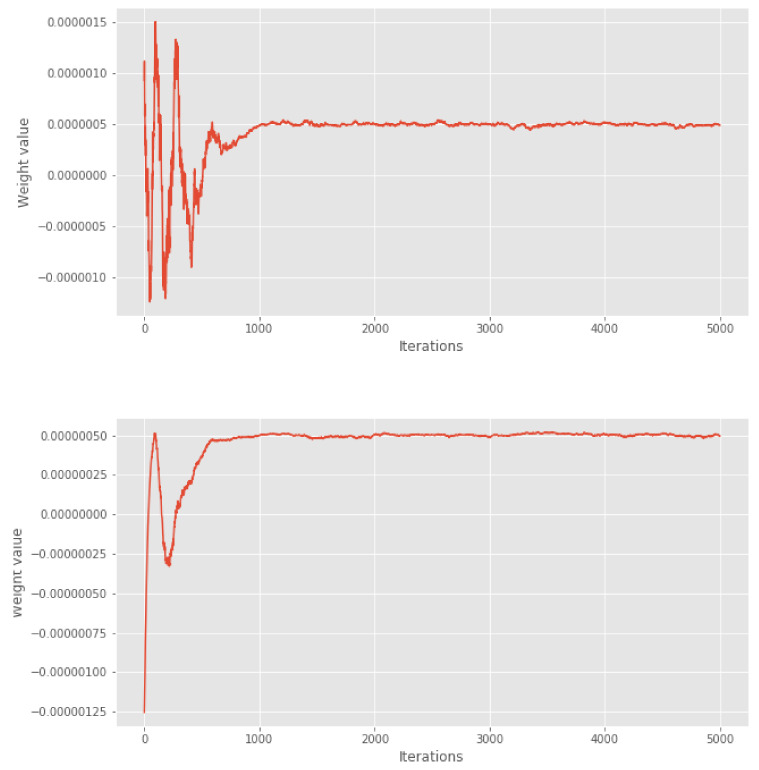
Lotka–Volterra example: first 5000 weights of the cube methods, based on full (**top**) or diagonal (**bottom**) set of covariates.

We note in passing that, in our experiments (see Figure 1 and the surrounding discussion), the weights $w_{n}$ make it easy to visually assess the convergence (and thus the burn-in) of the Markov chain. In fact, since the MCMC points of the burn-in phase are far from the mass of the target distribution, the procedure must assign a small or negative weight to these points in order to respect the constraints based on the control variates. Again, see Section 5.2 for more discussion on this issue. The fact that control variates may be used to assess MCMC convergence has been known for a long time (e.g., [16]), but the visualisation of weights makes this idea more expedient.

### 4.2. Second Step: Cube Resampling

The second step consists in resampling the weighted sample ${\left(w_{n},X_{n}\right)}_{n=1,\ldots ,N}$, to obtain a subsample $S=\{X_{n}:\;S_{n}=1\}$ where $S_{n}$ are random variables such that (a) $E\left[S_{n}\right]=w_{n}$; (b) $\sum_{n=1}^{N}S_{n}=M$, and (c) for j=1,…,J:

$$\sum_{S_{n}=1}h_{j}\left(X_{n}\right)=0.$$

Condition (a) ensures that the procedure does not introduce any bias:

$$E\left[\frac{1}{M}\sum_{S_{n}=1}f\left(X_{n}\right)|X_{1:N}\right]=\sum_{n=1}^{N}w_{n}f\left(X_{n}\right).$$

Condition (b) ensures that the subsample is exactly of size *M*.

We would like to use the cube method in order to generate the $S_{n}$’s. Specifically, we would like to assign the inclusion probabilities $\pi_{n}$ to $w_{n}$, and impose the (J+1) constraints defined above by conditions (b) and (c). There is one caveat, however: the weights $w_{n}$ do not necessarily lie in [0,1].

### 4.3. Dealing with Weights Outside of [0,1]

We rewrite (16) as:

(17) $${\hat{p}}_{⋆}\left(f\right)=\frac{\Omega }{M}\times \sum_{n=1}^{N}W_{n}\times \mathrm{sgn}\left(w_{n}\right)f\left(X_{n}\right)$$

where $\Omega =\sum_{n=1}^{N}\left|w_{n}\right|$ and $W_{n}=M\left|w_{n}\right|/\Omega$. We now have $W_{n}\geq 0$, and $\sum_{n=1}^{N}W_{n}=M$, which is required for condition (b) in the previous section. We might have a few points such that $W_{n}>1$. In that case, we replace them by $\left\lfloor W_{n}\right\rfloor$ copies, with adjusted weights $W_{n}/\left\lfloor W_{n}\right\rfloor$.

It then becomes possible to implement the cube method, using as inclusion probabilities the $W_{n}$s, and as the matrix *A* that defines the J+1 constraints, the matrix $A=(A_{jn})$ such that $A_{1n}=1$, $A_{(j+1)n}=\mathrm{sgn}\left(w_{n}\right)h_{j}\left(X_{n}\right)$. The cube method samples variables $S_{n}$, which may be used to compute the subsampled estimate

(18) $$\hat{\nu }\left(f\right)=\frac{\Omega }{M}\sum_{S_{n}=1}\mathrm{sgn}\left(w_{n}\right)f\left(X_{n}\right).$$

More generally, in our numerical experiments, we shall evaluate to which extent the random signed measure:

(19) $$\hat{\nu }=\frac{\Omega }{M}\sum_{S_{n}=1}\mathrm{sgn}\left(w_{n}\right)\delta_{X_{n}}\left(dx\right).$$

is a good approximation of the target distribution *p*.

## 5. Experiments

We consider two examples. The first example is taken from [5], and is used to compare cube thinning with KSD thinning. The second example illustrates cube thinning when used in conjunction with control variates that are not gradient-based. We also include standard thinning in our comparisons.

Note that there is little point in comparing these methods in terms of CPU cost, as KSD thinning is considerably slower than cube thinning and standard thinning whenever M≫100. (In one of our experiments, for M=1000, KSD took close to 7 h to run, while cube thinning with all the covariates took about 30 s.) Thus, our comparison will be in terms of statistical error, or, more precisely, in terms of how representative of *p* is the selected subsample.

In the following (in particular in the plots), “cubeFull” (resp. “cubeDiagonal”) will refer to our approach based on the full (resp. diagonal) set of control variates, as discussed in Section 2.3. “NoBurnin” means that burn-in has been discarded manually (hence, no burn-in in the inputs). Finally, “thinning” denotes the usual thinning approach, “SMPCOV”, “MED” and “SCLMED” are the same names used in [5] for KSD thinning, based on three different kernels.

To implement the cube method, we used R package BalancedSampling.

### 5.1. Evaluation Criteria

We could compare the three different methods in terms of variance of the estimates of p(f) for certain functions *f*. However, it is easy to pick functions *f* that are strongly correlated with the chosen control variates; this would bias the comparison in favour of our approach. In fact, as soon as the target is Gaussian-like, the control variates we chose in Section 2.3 should be strongly correlated with the expectation of any polynomial function of order two, as we discussed in that section.

Rather, we consider criteria that are indicative of the performance of the methods for a general class of function. Specifically, we consider three such criteria. The first one is the kernel Stein discrepency (KSD) as defined in [5] and recalled in the introduction—see (1). Note that this criterion is particularly favourable for KSD thinning, since this approach specifically minimises this quantity. (We use the particular version based on the median kernel in Riabiz et al. [5].)

The second criterion is the energy distance (ED) between *p* and the empirical distribution defined by the thinning method, e.g., (19) for cube thinning. Recall that the ED between two distributions *F* and *G* is:

(20) $$ED\left(F,G\right)=2E||Z-{X||}_{2}-E||Z-Z^{\prime }{\left|\right|}_{2}-E||X-X^{\prime }{\left|\right|}_{2}$$

where $Z^{\prime },Z\overset{iid}{∼}F$ and $X^{\prime },X\overset{iid}{∼}G$, and that this quantity is actually a pseudo-distance: ED(F,G)≥0, ED(F,G)=0⇒F=G, ED(F,G)=ED(G,F), but ED does not fulfil the triangle inequality [17,18].

One technical difficulty is that (19) is a signed measure, not a probability measure; see Appendix B on how we dealt with this issue.

Our third criterion is inspired by the star discrepancy, a well-known measure of the uniformity of *N* points $u_{n}\in {\left[0,1\right]}^{d}$ in the context of quasi-Monte Carlo sampling [9] (Chapter 15). Specifically, we consider the quantity

$$d^{⋆}\left(\hat{P},\hat{\nu }\right)=\underset{B\in B}{\sup}\left|{\hat{P}}_{\psi }\left(B\right)-{\hat{\nu }}_{\psi }\left(B\right)\right|$$

where $\psi :R^{d}\to {\left[0,1\right]}^{d}$, ${\hat{P}}_{\psi }$ and ${\hat{\nu }}_{\psi }$ are the push-forward measures associated to empirical distributions $\hat{P}={\left(N-b\right)}^{-1}\sum_{n=b+1}^{N}\delta_{X_{n}}\left(dx\right)$, and $\hat{\nu }$ as defined in (19), and B is the set of hyper-rectangles $B=\prod_{i=1}^{d}\left[0,b_{i}\right]$. In practice, we defined function ψ as follows: we apply the linear transform that makes the considered sample to have zero mean and unit variance, and then we applied the inverse CDF (cumulative distribution function) of a unit Gaussian to each component.

Additionally, since the sup above is not tractable, we replace it by a maximum over a finite number of $b_{i}$ (simulated uniformly).

### 5.2. Lotka–Volterra Model

This example is taken from [5]. The Lotka–Volterra model describes the evolution of a prey–predator system in a closed environment. We denote the number of prey by $u_{1}$ and the number of predators by $u_{2}$. The growth rate of the prey is controlled by a parameter $\theta_{1}>0$ and its death rate—due to the interactions with the predators—is controlled by a parameter $\theta_{2}>0$. In the same way, the predator population has a death rate of $\theta_{3}>0$ and a growth rate of $\theta_{4}>0$. Given these parameters, the evolution of the system is described by a system of ODEs:

$$\begin{array}{cc} \frac{du_{1}}{dt}= & \theta_{1}u_{1}-\theta_{2}u_{1}u_{2}\\ \frac{du_{2}}{dt}= & \theta_{4}u_{1}u_{2}-\theta_{3}u_{2} \end{array}$$

Ref. [5] set $\theta =\left(\theta_{1},\theta_{2},\theta_{3},\theta_{4}\right)=\left(0.67,1.33,1,1\right)$, the initial condition $u_{0}=\left(1,1\right)$, and simulate synthetic data. They assume they observe the populations of prey and predator at times $t_{i},i=1,\cdots ,2400$ where the $t_{i}$ are taken uniformly on [0,25] and that these observations are corrupted with a centered Gaussian noise with a covariance matrix $C=\mathrm{diag}(0.2^{2},0.2^{2})$. Finally, the model is parametrised in terms of $x=\left(\log\theta_{1},\log\theta_{2},\log\theta_{3},\log\theta_{4}\right)\in R^{4}$ and a standard normal distribution as a prior on *x* is used.

The authors have provided their code as well as the sampled values they obtained by running different MCMC chains for a long time. We use the exact same experimental set-up, and we do not run any MCMC chain on our own, but use the ones they provide instead, specifically the simulated chain, of length $2\times 10^{6}$, from preconditionned MALA.

We compress this chain into a subsample of size either M=100 or M=1000. For each value of *M*, we run different variations of our cube method 50 times and make a comparison with the usual thinning method and with the KSD thinning method with different kernels, see [5]. In Figure 1, we show the first 5000 weights of the cube method. We can see that after 1000 iterations, the weights seem to stabilise. Based on visual examination of these weights, we chose a conservative burn-in period of 2000 iterations for the variants where burn-in is removed manually.

**Figure 2:**
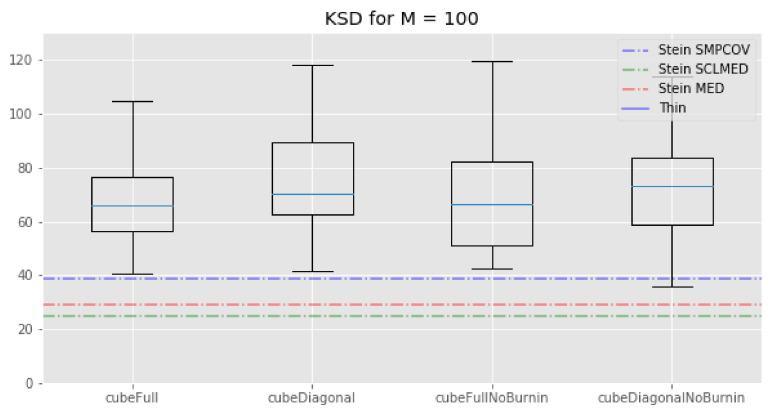

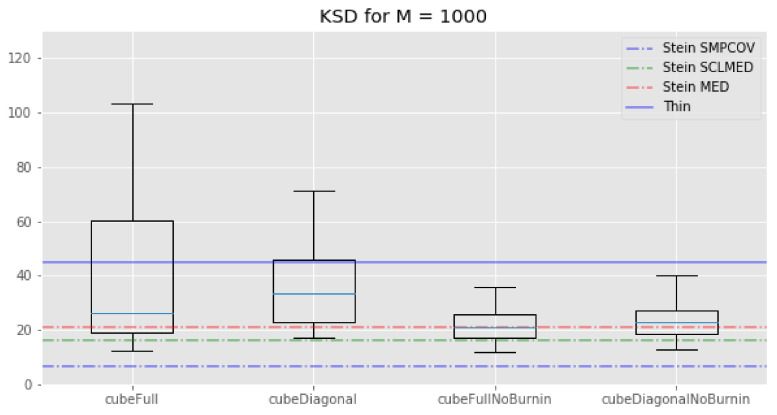
Lotka–Volterra example: box-plots of the kernel Stein discrepency for all the cube method variations, compared with the KSD method for three kernels and the usual thinning method (horizontal lines). **Top**: M=100. **Bottom**: M=1000. (In the top plot, standard thinning is omitted to improve clarity, as corresponding value is too high.)

**Figure 3:**
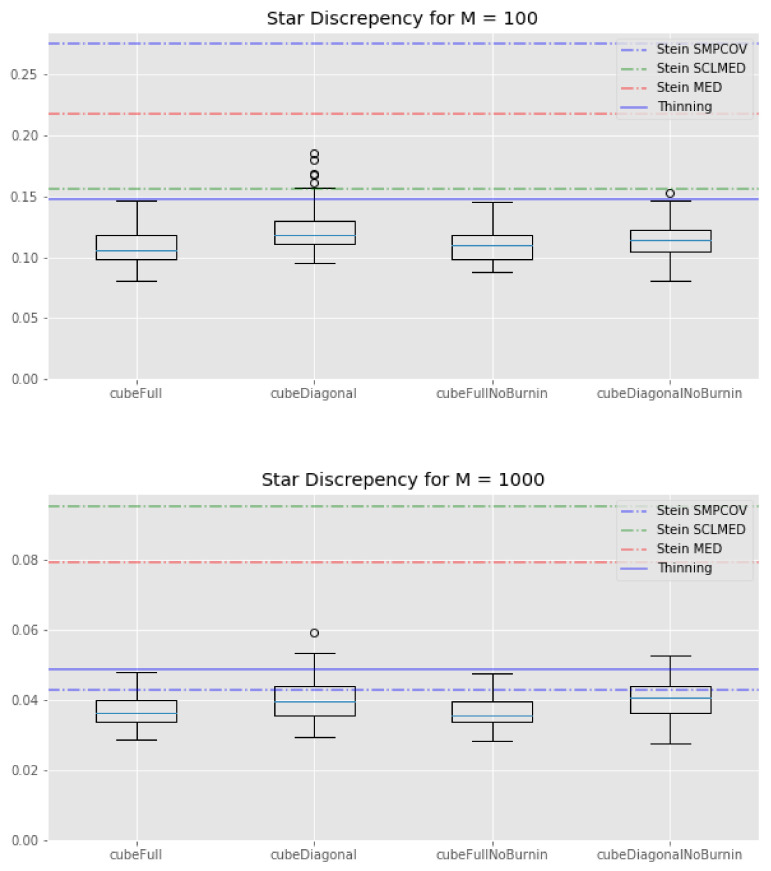
Lotka–Volterra example: box-plots of the star discrepency for all the cube method variations, compared with the KSD method for three kernels and the usual thinning method (horizontal lines). **Top**: M=100. **Bottom**: M=1000.

**Figure 4:**
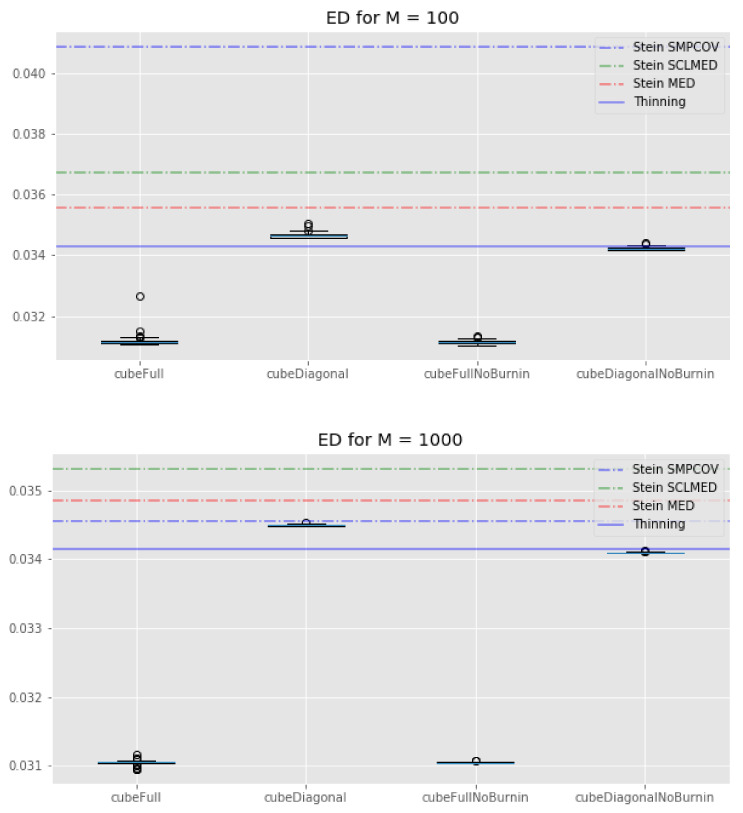
Lotka–Volterra example: boxplots of the energy distance for all the cube method variations, compared with the KSD method for three kernels and the usual thinning method (horizontal lines). **Top**: M=100. **Bottom**: M=1000.

We plot the results of the experiment in Figure 2, Figure 3 and Figure 4.

First, we see that regarding the kernel Stein discrepancy metric, Figure 2, the KSD method performs better than the standard thinning procedure and the cube method. This is not surprising since, even if this method does not properly minimise the Kernel–Stein Discrepency, this is still its target. We also see that, for M=1000, the KSD method performs a bit better than our cube method which in turn performs better than the standard thinning procedure. Note that the relative performance of the KSD method to our cube methods depends on the kernel that is being used and that there is no way to determine which kernel will perform best before running any experiment.

The picture is different for M=100: KSD thinning outperforms standard thinning, which in turn outperforms all of our cube thinning variations. Once again, the fact that the KSD method performs better than any other method seems reasonable: since it regards minimizing the Kernel–Stein Discrepancy, the KSD method is “playing at home” on this metric.

If we look at Figure 4, we see that all of our cube methods outperform the KSD method with any kernel. Interestingly, the standard thinning methods has a similar energy distance as the cube methods with “diagonal” control variates. These observations are true for both M=100 and M=1000. We can also note that the cube method with the full set of control variates tends to perform much better than its “diagonal” counterpart, whatever the value of *M*.

Finally, looking at Figure 3, it is clear that the KSD method—with any kernel—performs worse than any cube method in terms of star discrepancy.

Overall, the relative performance of the cube methods and KSD methods can change a lot depending on the metric being used and the number of points we keep. In addition, while all the cube methods tend to perform roughly the same, this is not the case of the KSD method, whose performances depend on the kernel we use. Unfortunately, we have no way to determine beforehand which kernel will perform best. This is a problem since the KSD method is computationally expensive for subsamples of cardinality M≫100.

Thus, by and large, cube thinning seems much more convenient to use (both in terms of CPU time and sensitivity to tuning parameters) while offering, roughly, the same level of statistical performance.

### 5.3. Truncated Normal

In this example, we use the (random-scan version of) the Gibbs sampler of [1] to sample from 10-dimensional multivariate normal truncated to ${\left[0,\infty \right)}^{10}$. We generated the parameters of this truncated normal as follows: the mean was set as the realisation of a 10-dimensional standard normal distribution, while for the covariance matrix Σ, we first generated a matrix $M\in M_{10,10}\left(R\right)$ for which each entry was the realisation of a standard normal distribution. Then, we set $\Sigma =M^{T}M$.

Since we used a Gibbs sampler, we have access to the Gibbs control variates of [7], based on the expectation of each update (which amounts to simulating from a univariate Gaussian). Thus, we consider 10 control variates.

The Gibbs sampler was run for $N=10^{5}$ iterations and no burn-in was performed. We compare the following estimators of the expectation of the target distribution the standard estimator, based on the whole chain ("usualEstim" in the plots), the estimator based on standard thinning ("thinEstim" in the plots), the control variate estimator based on the whole chain, i.e., (7) ("regressionEstim" in the plots), and finally our cube estimator described in Section 4 ("cubeEstim" in the plots). For standard thinning and cube thinning, the thinning sample size was set to M=100, which corresponds to a compression factor of $10^{3}$.

**Figure 5:**
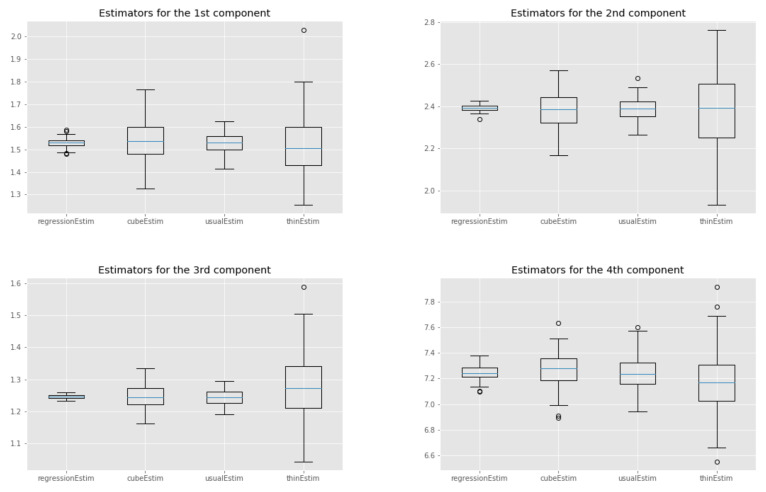
Truncated normal example: box-plots over 100 independent replicates of each estimator; see text for more details.

The results are shown in Figure 5. First, we can see that the control variates we chose led to a substantial decrease in the variance of the estimates for regressionEstim compared to usualEstim. Second, the cube estimator performed worse than the regression estimator in terms of variance, but this was expected, as explained in Section 4. More interestingly, if we cannot say that the cube estimator performs better than the usual MCMC estimator in general, we can see that for some components it performed as well or even better, even though the cube estimator used only M=100 points while the usual estimator used $10^{5}$ points. This is largely due to the good choice of the control variates. Finally, the cube estimator outperformed the regular thinning estimator for every component, sometimes significantly.

## Data Availability

The data that support the findings of the first numerical experiment are openly available in stein.thinning at https://github.com/wilson-ye-chen/stein.thinning (accessed on 2 August 2021).

## Appendix A. Details on the Landing Phase

The landing phase seeks to generate a random vector *S* in ${\left\{0,1\right\}}^{N}$, with expectation $\pi^{⋆}$ (the output of the flight phase), which minimises the criterion $tr(M\mathrm{Var}\left(\hat{V}|\pi^{⋆}\right))$ for a certain matrix *M*. (The notation $\cdot |\pi^{⋆}$ refers to the distribution of *S* conditional on $\pi \left(t\right)=\pi^{⋆}$ at the end of the flight phase.)

Since $\mathrm{Var}\left(S\right)=\mathrm{Var}\left(E\left[S|\pi^{⋆}\right]\right)+E\left[\mathrm{Var}\left(S|\pi^{⋆}\right)\right]$ by the law of total variance, and since the first term is zero (as $E\left[S|\pi^{⋆}\right]=\pi^{⋆}$), we have

(A1) $$\mathrm{Var}\left(\hat{V}\right)=E\left[\mathrm{Var}\left(\hat{V}|\pi^{⋆}\right)\right]=E\left[A\mathrm{Var}\left(S|\pi^{⋆}\right)A^{t}\right].$$

and thus:

(A2) $$\mathrm{tr}\left(M\mathrm{Var}\left(\hat{V}|\pi^{⋆}\right)\right)=\sum_{s\in {\left\{0,1\right\}}^{N}}p\left(s|\pi^{⋆}\right){\left(s-\pi^{⋆}\right)}^{t}A^{t}MA\left(s-\pi^{⋆}\right).$$

Choosing $M={\left(AA^{t}\right)}^{-1}$, as recommended by [6], amounts to minimising the distance to the hyperplane ‘on average’. Let $C\left(s\right)={\left(s-\pi^{⋆}\right)}^{t}A^{t}{\left(AA^{t}\right)}^{-1}A^{t}\left(s-\pi^{⋆}\right)$, then the minimisation program is equivalent to the following linear programming problem over *q* variables only:

(A3) $$\min_{\xi^{⋆}\left(.\right)}\sum_{s^{⋆}\in S^{⋆}}C\left(s^{⋆}\right)\xi^{⋆}\left(s^{⋆}\right)$$

with constraints $\sum_{s^{⋆}\in S^{⋆}}\xi^{⋆}\left(s^{⋆}\right)=1$, $0\leq \xi^{⋆}\left(s^{⋆}\right)\leq 1$, $\sum_{s^{⋆}\in S^{⋆}|s_{k}^{⋆}=1}\xi^{⋆}\left(s^{⋆}\right)=\pi_{k}^{⋆}$ for every $k\in U^{⋆}$ and $S^{⋆}={\left\{0,1\right\}}^{q}$ where $q=\mathrm{card}(U^{⋆})$ and $U^{⋆}=\left\{k\in U:\;0<\pi^{⋆}\left[k\right]<1\right\}$. Here, $\xi^{⋆}$ denotes the marginal distribution of the components $U^{⋆}$ of the sampling design ξ and $C(s^{⋆})$ must be understood as C(s) with the components of $s\notin U^{⋆}$ being fixed by the result of the flight phase; thus, in this minimisation problem, *C* is in fact dependent on the components of *s* that are in $U^{⋆}$ only.

The constraints define a bounded polyhedron. By the fundamental theorem of linear programming, this optimisation problem has at least one solution on a minimal support—see [6].

The flight phase ends on a vertex of K and, by Proposition 1 in [6], q≤J—typically J≪N. This means that we are solving a linear programming problem in a dimension *q* potentially much lower than the population size *N*, and if we do not have too many auxiliary variables, this optimisation problem will not be computationally too expensive. In practice, a simplex algorithm is used to find the solution.

## Appendix B. Estimation of the Energy Distance

There are two difficulties with computing (20). First, it involves intractable expectations. Second, as pointed out at the end of Section 4.3, the empirical distribution generated by cube thinning, (19), is actually a signed measure.

Regarding the first issue, we can approximate (20) from our MCMC sample $X_{1},\cdots ,X_{N}$. That is, if our subsampled empirical measure writes $\hat{\nu }=\sum_{m=1}^{M}w_{m}\delta_{Z_{m}}$ and that we approximate the distribution associated with *p* by $\hat{P}={\left(N-b\right)}^{-1}\sum_{n=b+1}^{N}\delta_{X_{n}}$ where 1≤b≤N is the burn-in of the chain; then, we can estimate $ED(\hat{\mu },p)$ with $ED(\hat{\mu },\hat{P})$.

Regarding the second issue, we can generalize the energy distance to finite measures: suppose we have two finite and potentially signed measures $\nu_{1}$ and $\nu_{2}$, both defined on the same measurable space (Ω,P(Ω)} where $\Omega =\{X_{1},\cdots ,X_{N}\}$ and P(Ω) denote the set of parts of Ω. Suppose, in addition, that $\nu_{1}\left(\Omega \right)=\alpha_{1}$ and $\nu_{2}\left(\Omega \right)=\alpha_{2}$ with $\alpha_{1}\neq 0$ and $\alpha_{2}\neq 0$. We define the generalized energy distance as:

$$\begin{array}{cc} ED^{⋆}\left(\nu_{1},\nu_{2}\right)=\; & \frac{2}{\alpha_{1}\alpha_{2}}\int_{\Omega }||x-{y||}_{2}d\nu_{1}\left(x\right)d\nu_{2}\left(y\right)\\ \; & -\frac{1}{\alpha_{1}^{2}}\int_{\Omega }||x-x^{\prime }{\left|\right|}_{2}d\nu_{1}\left(x\right)d\nu_{1}\left(x^{\prime }\right)\\ \; & -\frac{1}{\alpha_{2}^{2}}\int_{\Omega }||y-y^{\prime }{\left|\right|}_{2}d\nu_{2}\left(y\right)d\nu_{2}\left(y^{\prime }\right). \end{array}$$

Then, by negative definiteness of the application $\phi \left(x,y\right)=||x-{y||}_{2}$ on $R^{N}\times R^{N}$, $ED^{⋆}\left(\nu_{1},\nu_{2}\right)\geq 0$ with equality if and only if $\frac{1}{\alpha_{1}}\nu_{1}=\frac{1}{\alpha_{2}}\nu_{2}$. This means that the generalized energy distance is zero if and only if the two measures are equal up to a non-zero multiplicative constant—see [17] for a demonstration. This generalized energy distance is also symmetric, but the triangle inequality does not hold. It is a pseudo-distance.

Thus, we will use the following criterion, which we will call the energy distance:

$$\begin{array}{cc} {ED}^{⋆}\left(\hat{\nu },\hat{P}\right)= & \frac{2}{\left(N-b\right)\alpha_{1}}\sum_{k=1}^{N}\sum_{n=b+1}^{N}\frac{\Omega }{M}sgn\left(w_{k}\right)\left|\right|X_{k}-X_{n}{\left|\right|}_{2}1_{\left\{S_{k}=1\right\}}\\ \; & -\frac{1}{\alpha_{1}^{2}}\sum_{n=1}^{N}\sum_{k=1}^{N}{\left(\frac{\Omega }{M}\right)}^{2}sgn\left(w_{n}\right)sgn\left(w_{k}\right)\left|\right|Z_{k}-Z_{n}{\left|\right|}_{2}1_{\left\{S_{k}=1\right\}}1_{\left\{S_{n}=1\right\}} \end{array}$$

where $\hat{\nu }$ is defined in (19) and we dropped the last term because it does not depend on $\hat{\nu }$ and it is a potentially expensive sum of ${\left(N-b\right)}^{2}$ terms.

Note that the probability of $\hat{\nu }\left(\Omega \right)$ being zero is non-null and then there is a non-negligible probability of ${ED}^{⋆}\left(\hat{\nu },\hat{P}\right)$ being undefined. However, this event is unlikely to happen.
